# Interactive cost of *Plasmodium* infection and insecticide resistance in the malaria vector *Anopheles gambiae*

**DOI:** 10.1038/srep29755

**Published:** 2016-07-19

**Authors:** Haoues Alout, Roch K. Dabiré, Luc S. Djogbénou, Luc Abate, Vincent Corbel, Fabrice Chandre, Anna Cohuet

**Affiliations:** 1 Institut de recherche pour le développement (IRD), Maladies Infectieuses et Vecteurs, Ecologie, Génétique, Evolution et Contrôle (MIVEGEC), UM1-UM2-CNRS 5290 IRD 224, Montpellier, France; 2 Institut de Recherche en Sciences de la Santé (IRSS), 01 BP 545 Bobo-Dioulasso 01, Burkina Faso; 3 Institut Régional de Santé Publique/Université d’Abomey-Calavi, 01 BP 918 Cotonou, Bénin; 4 Department of Entomology, Faculty of Agriculture, Kasetsart University, Bangkok 10900, Thailand

## Abstract

Insecticide resistance raises concerns for the control of vector-borne diseases. However, its impact on parasite transmission could be diverse when considering the ecological interactions between vector and parasite. Thus we investigated the fitness cost associated with insecticide resistance and *Plasmodium falciparum* infection as well as their interactive cost on *Anopheles gambiae* survival and fecundity. In absence of infection, we observed a cost on fecundity associated with insecticide resistance. However, survival was higher for mosquito bearing the *kdr* mutation and equal for those with the *ace-1*^*R*^ mutation compared to their insecticide susceptible counterparts. Interestingly, *Plasmodium* infection reduced survival only in the insecticide resistant strains but not in the susceptible one and infection was associated with an increase in fecundity independently of the strain considered. This study provides evidence for a survival cost associated with infection by *Plasmodium* parasite only in mosquito selected for insecticide resistance. This suggests that the selection of insecticide resistance mutation may have disturbed the interaction between parasites and vectors, resulting in increased cost of infection. Considering the fitness cost as well as other ecological aspects of this natural mosquito-parasite combination is important to predict the epidemiological impact of insecticide resistance.

Malaria is a parasitic disease caused by protozoa of the genus *Plasmodium*, which are transmitted between vertebrate hosts through mosquito bites. In 2015, 214 million cases occurred with approximately 438,000 deaths, ~70% of which were children younger than five years of age^1^. To combat malaria, current strategies include anti-malarial drug treatments alongside vector control using long-lasting insecticidal bed-nets and indoor-residual spraying of insecticides. However, widespread insecticide resistance in vectors^2^ and concern over spreading artemisinin resistance^3^,^4^ trigger the fragility of malaria treatment and control. To assess the epidemiological impact of such adaptation, it is crucial to understand the ecological processes underlying the co-evolution of vector-parasite interactions. Genes responsible for an adaptation to a new environment are usually assumed to be detrimental in the previous environment^5^. The new adaptive allele may be associated with modification of physiological processes or resource availability, which often leads to decrease performance and overall fitness^6^,^7^. Such a fitness cost is a determinant factor in the evolution of an adaptation and has been well characterized for insecticide resistance in arthropods species^8^,^9^. Unraveling these costs on natural vector-parasite interactions is essential for guiding the implementation of vector control strategies^10^.

As a result of an intense selective pressure from insecticides to reduce vector longevity and abundance, several resistance mechanisms have been selected in mosquito vectors^11^. Among them two main mechanisms are responsible for high level of resistance: increased metabolism of detoxification (named metabolic resistance) and modification of the insecticide target site (named target-site resistance)^11^. The molecular basis of target-site resistance has been characterized in many insect species and has demonstrated conserved resistant mutations^11^. In several mosquito species and populations, the unique G119S mutation in the *ace-1* gene (*ace-1*^*R*^ allele) is responsible for organophosphate and carbamate resistance^12^, the L1014F mutation in the *para*-type sodium channel gene (*kdr*-west mutation) is responsible for pyrethroid resistance in malaria vectors from West Africa^13^, and the A302G mutation in the *Rdl* gene in *An. gambiae* is responsible for cyclodiene resistance^14^. In mosquitoes, resistance alleles spread and increase in frequency in insecticide-treated areas whereas in non-treated areas their frequency remains low, thus revealing a substantial fitness cost^15^. The fitness cost associated with insecticide resistance has been characterized on a large range of life history traits: such as development time, mortality and predation avoidance in larvae, flight and host-seeking activities, male reproductive success, fecundity, survival and susceptibility to infection in adults^8^,^16^,^17^,^18^,^19^,^20^,^21^,^22^,^23^,^24^,^25^. It remains however uncertain to which extend insecticide resistance would have an impact on vector population dynamics and parasite transmission^26^.

Another fitness cost that may have great impact on the coevolutionary dynamic of mosquito and parasite might be the cost that the parasite may impose to its own vector. Investigation of the fitness cost of *Plasmodium* infection in vector mosquitoes resulted in conflicting results. Studies carried out on non-natural vector-parasite combinations mainly showed that infection affects mosquito fitness related traits such as survival and fecundity, while studies of natural combinations of vector and parasites revealed more complex results^27^. Cost of infection in the natural vectorial system responsible for transmission of human malaria *An. gambiae-P. falciparum* was not detected in some studies^28^,^29^ whereas cost on fecundity^30^ and stress-dependent cost on survival was observed upon infection in other studies^31^,^32^. In the natural avian malaria system, *Culex pipiens-P. relictum*, a decreased fecundity and an increased survival was observed^33^ as well as a stress-dependent cost on survival^34^,^35^. These studies therefore showed that *Plasmodium* infection is more likely to imply a cost when mosquitoes experience unfavorable environmental conditions.

Most research has focused on the direct consequences of mosquito adaptation to the insecticides to assess the entomological efficacy of vector control, but they do not necessarily take into account the ecological complexity of the natural vectorial systems in order to predict its epidemiological impact. However, complex interactions have been recently identified. For instance, insecticide resistant mutations have been associated with increased vector competence in absence of insecticides^22^. In contrast, exposure to insecticide decrease the competence of resistant vectors for malaria parasite^36^. In addition, *P. falciparum* infection reduces the ability of resistant vectors to survive insecticide exposure^37^. This illustrates complex interactions between insecticide resistance, exposure to insecticide and malaria parasite transmission. However, to our knowledge, it remains unknown whether cost of infection and cost of insecticide resistance may interplay in malaria vectors.

Therefore, in this study, we aim to determine the cost of insecticide resistance and its impact on the cost of *Plasmodium* infection. We measured the survival and fecundity of *Anopheles gambiae s.s.* either susceptible or harboring the resistant *kdr* or *ace-1*^*R*^ mutations (target site resistance mechanism) in absence of insecticides. The strains shared a common genetic background to avoid any confounding effect due to other resistance mechanisms or any other mutations that are not in linkage disequilibrium. We compared these life history traits between *Plasmodium* infected and unexposed mosquitoes from each strains and demonstrated that the cost of infection is higher in the resistant vectors than in the susceptible ones.

## Results

We performed experimental infections of laboratory strains of *Anopheles gambiae s.s.* with wild isolates of *Plasmodium falciparum* using the standard membrane feeding assay protocol^38^ implemented by Sangaré *et al*.^39^ to obtain the corresponding non-infectious control blood. Five replicates each with a distinct blood donor were performed for each experiment (survival and fecundity, respectively). The proportion of infected mosquitoes among those that took an infectious blood meal (all experiments included) varied from 51.4% to 97.6% for Kisumu, from 58.1% to 96.9% for Acerkis, and from 61% to 100% for Kdrkis strain. Consistent with previous data^22^, the susceptible strain Kisumu showed the lowest susceptibility to infection (averaged across replicates, 66.4% ± 2.3) and the resistant strains were more susceptible to *Plasmodium* infection (Acerkis: 79.7% ± 2.0, *p* < 0.001 and Kdrkis: 80.2% ± 2.0, *p* < 0.001).

### Survival

We followed 1290 female mosquitoes of the three strains from blood feeding until death, among them 649 took a gametocyte-infectious blood meal and 641 took a heat inactivated (control) blood meal through five replicates. Among the 649 blood-fed females on infectious blood, 487 were oocyst-infected after midgut dissection; the females that did not carry oocysts were removed from the following analysis. Cox proportional analysis of the survivorship on the whole data set indicated that the blood donor has significantly influenced mosquito survivorship (variance: 0.08459 ± 0.06319). The analysis revealed also a significant impact of the *strain* (χ^2^_df=2_ = 30.38, *p* < 0.001), the *infection* status (χ^2^_df=1_ = 27.90, *p* < 0.001) and the *strain* by *infection* interaction (χ^2^_df=2_ = 13.43 *p* = 0.0012) but *heamatin* (indicative of blood meal size) and *wing length* (proxy of body size) and their interactions were not retained in the minimal model.

#### Influence of insecticide resistance

**Figure 1:**
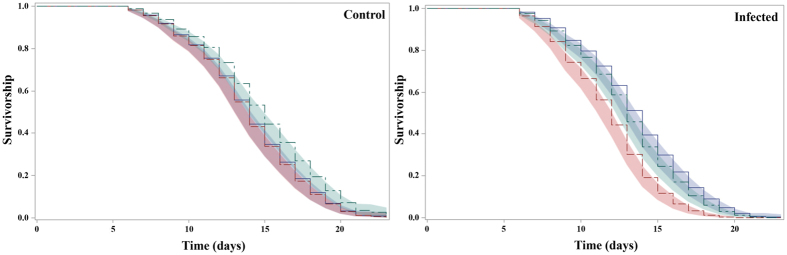
Kaplan-Meier survival curves of the susceptible strain Kisumu (blue) the resistant strains Acerkis (red) and Kdrkis (green) within control or infected mosquitoes. The control group corresponds to mosquitoes that fed on non-infectious blood and the infected group to mosquitoes with at least one oocyst in the midgut.

Figure 1 presents the Kaplan-Meier survival curves for each strain within each infection status (*Plasmodium* infected or control mosquitoes). The survival distributions were significantly different between mosquito strains in the control (*i.e.* unexposed to infection) (Log-Rank statistic, χ^2^_df=2_ = 14.48, *p* < 0.001) or in the infected group (Log-Rank statistic, χ^2^_df=2_ = 32.86, *p* < 0.001). Interestingly, comparison among mosquito strains unexposed to infection revealed a similar survivorship between Acerkis and Kisumu (HR = 0.96, Confidence Interval, CI: [0.78–1.17], Table 1) but a higher survivorship of Kdrkis over that of Kisumu (HR = 1.37, CI: [1.12–1.68]). However, among *Plasmodium* infected mosquitoes, Acerkis females had a lower survivorship than Kisumu (HR = 0.55, CI: [0.44–0.69]), while survivorship of Kdrkis and Kisumu were similar (HR = 0.90, CI: [0.72–1.12]).

#### Influence of P. falciparum infection

**Figure 2:**
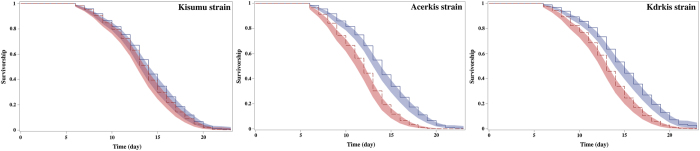
Kaplan-Meier survival curves of infected (red) and control (blue) mosquitoes within susceptible (Kisumu) or resistant (Acerkis and Kdrkis) strains of *An. gambiae.* For each strain, the control group corresponds to mosquitoes that fed on non-infectious blood and the infected group to mosquitoes with at least one oocyst in the midgut.

Figure 2 presents the Kaplan-Meier survival curves for control (non-exposed to *Plasmodium*) and infected mosquitoes within each strain. Infection did not impact survival of the susceptible Kisumu strain (Log-Rank statistic, χ^2^_df=1_ = 1.54, *p* = 0.21, Table 1). However survival analysis on resistant mosquitoes, separately, indicated a significant difference between infected and control groups (Log-Rank statistic, χ^2^_df=1_ = 49.45, *p* < 0.001 for Acerkis and χ^2^_df=1_ = 39.26, *p* < 0.001 for Kdrkis), revealing a cost of *P. falciparum* infection only in insecticide resistant mosquitoes. Survivorship of the susceptible strain Kisumu did not differ significantly between *Plasmodium* infected and unexposed mosquitoes (HR = 0.90, CI: [0.72–1.12], Table 1). However *Plasmodium* infection reduced the survivorship in both resistant strains: Acerkis and Kdrkis infected females had about twice less chance to survive at any time than the unexposed females (HR = 0.52, CI: [0.42–0.64] and 0.59, CI: [0.48–0.71] for Acerkis and Kdrkis respectively).

### Fecundity

Female mosquitoes of the three strains were allowed to lay eggs individually after blood feeding on infectious or non-infectious control blood (1BM, N = 688). Statistical analysis of the fecundity rate revealed a significant influence of insecticide resistant alleles (*strain* variable: F_df=2,677_ = 4.96, *p* = 0.007), the infection status (F_df=1,677_ = 9.14, *p* = 0.002) as well as the interaction of insecticide resistance and body size (*strain:wing*: F_df=2,677_ = 4.81, *p* = 0.008) indicating a distinct impact of body size depending in the different strains. Among females that have laid at least one egg (N = 271), only the interaction between *wing length* and insecticide resistance *strains* has significantly influenced the number of eggs (*wing:strain* interaction: F_df=2,261_ = 5.13, *p* = 0.006).

#### Influence of insecticide resistance

**Figure 3:**
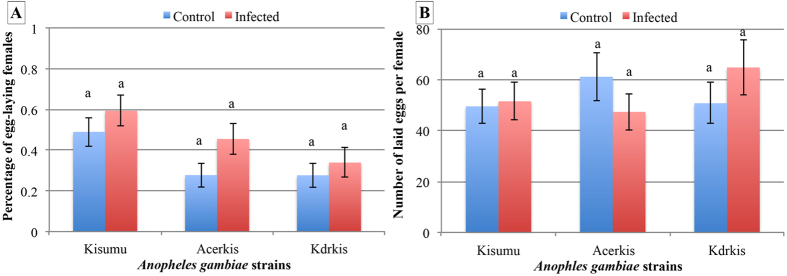
Fecundity of infected and control mosquitoes for each *Anopheles gambiae* strain following a single blood meal. Panel A presents the percentage of females producing eggs and panel B presents the number of eggs per females. Red bars represent infected females and blue bars represent females unexposed to *P. falciparum*. Error bars represent the standard error of the mean.

In absence of *Plasmodium* infection, both insecticide resistant strains had a lower fecundity rate than Kisumu (27.8% ± 5.9 for Acerkis and 27.7% ± 6.0 for Kdrkis compared to 49.0% ± 7.2 for Kisumu, *p* < 0.001, Table 2 and Fig. 3A). When comparing infected individuals, only the Kdrkis strain showed a lower fecundity rate compared to the susceptible strain Kisumu (33.9% ± 7.3 for Kdrkis compared to 59.4% ± 7.5 for Kisumu, *p* = 0.002; and 45.5% ± 7.5 for Acerkis, *p* = 0.065). While wing length did not affect fecundity rate in the susceptible strain, we observed an opposite relationship in the two insecticide resistant strains: in Acerkis smaller females had a greater probability to produce eggs while in Kdrkis bigger female had a greater probability to produce eggs. When comparing females that produced at least one egg (Fig. 3B), no difference in the number of laid eggs wa oserved between mosquito strains. However, the influence of body size on the number of eggs depended on the strains: in the Kdrkis strain, bigger females produced more eggs whereas no correlation was observed in the Kisumu and Acerkis strains.

#### Influence of P. falciparum infection

Overall, *Plasmodium* infection increased the fecundity rate of *An. gambiae*, regardless of the strain (Control: 34.8% ± 6.4 vs. infected: 44.6% ± 7.4, *p* = 0.003). When the difference in fecundity rate was tested separately within each strain (Table 2, Fig. 3A), the difference between infected and unexposed control was not significant (Kisumu: *p* = 0.152; Acerkis: *p* = 0.055; Kdrkis: *p* = 0.345).

### Fecundity of pre-gravid female

In the field, *An. gambiae s.l.* commonly take two blood meals before they lay their first batch of eggs probably due to insufficient resources for egg maturation^40^,^41^. Thus the female mosquitoes that did not produce eggs following the first blood meal were allowed to re-blood feed on rabbit blood and isolated again to lay eggs (N = 547). The analysis of the fecundity rate among these pre-gravid females demonstrated a significant influence of insecticide resistance alleles (*strain:* F_df=2,537_ = 4.04, *p* = 0.018) and the interaction between the strains and body size (*strain:wing* interaction: F_df=2,537_ = 4.56, *p* = 0.011). Among the females that have laid at least one egg (N = 300), the number of eggs was significantly influenced by *Plasmodium* infection (*status*: F_df=1,292_ = 5.45, *p* = 0.006) and by the interaction between body size and infection (*wing:status* interaction: F_df=1,292_ = 4.31, *p* = 0.039).

#### Influence of insecticide resistance

**Figure 4:**
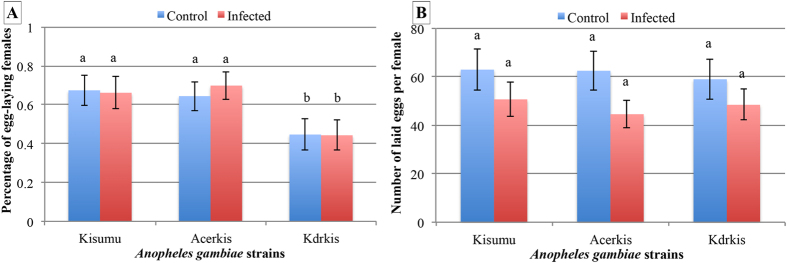
Fecundity of infected and control mosquitoes for each *Anopheles gambiae* strain following a second blood meal to complete their first gonotrophic cycle. Panel A presents the percentage of females producing eggs and panel B presents the number of eggs per females. Red bars represent infected females and blue bars represent females unexposed to *P. falciparum*. Error bars represent the standard error of the mean.

Fecundity rates were significantly lower in Kdrkis females compared to Kisumu (control: 45.5% ± 8.2 vs. 66.8% ± 8.0, p = 0.013; infected: 44.2% ± 7.6 vs. 65.7% ± 78.1, p = 0.008, Table 2, Fig. 4A). No differences were observed between the OP resistant strain Acerkis and the susceptible strain Kisumu (control: 64.4% ± 7.4 vs. 66.8% ± 8.0, p = 0.762; infected: 70.6% ± 7.0 vs. 65.7% ± 8.1, p = 0.518). In addition, no influence of insecticide resistance on the number of eggs produced was observed neither in infected nor in control mosquitoes (Table 2, Fig. 4B).

#### *Influence of* P. falciparum *infection*

No effect of infection was observed on the fecundity rate of females that needed 2 blood meals to complete egg maturation. However, in presence of *Plasmodium* infection, the number of eggs produced was reduced, regardless of the strain (Control: 61.8 ± 6.5 vs. infected: 47.5 ± 5.0, p = 0.0006). In addition, bigger females produced fewer eggs in contrast to the unexposed females, for which there was no influence of the body size.

## Discussion

In order to determine the cost of insecticide resistance mutations in *An. gambiae s.s.*, we compared the survival and fecundity of strains with a common genetic background in absence of insecticides. In addition, we used natural isolates of *P. falciparum* to determine the cost of infection in susceptible and resistant *An. gambiae*. To this aim, we compared the studied life history traits between gametocyte-exposed, infected mosquito and non-exposed control counterparts but we did not include mosquito exposed and not infected as their number were limited.

We assumed that variation in the life history traits between the insecticide susceptible and the resistant strains are associated with the insecticide resistance alleles or with any tightly linked loci resulting from hitchhiking during introgression. Indeed, the genetic differences between the susceptible and the resistant strains might not be totally negligible: a portion of ~11 Mb around the resistant allele may remain different between strains after 19 backcrosses^17^,^42^. It was for instance hypothesized that the increased competence for *Plasmodium* parasite in insecticide resistant mosquitoes^22^,^43^ was caused by a predicted immune gene closely linked to the *kdr* mutation^44^. Then the causative mutations would be also highly linked to insecticide resistance in natural populations. Consistent to published results^44^, we found polymorphism on SNPs linked to the *para* locus in the susceptible strain Kisumu while in the resistant Kdrkis, all individuals shared the same haplotype (*i.e.* no polymorphism on the tested SNPs, supplementary information). Therefore, we cannot exclude the possibility that the observed differences between the two strains are derived from genetic variance rather than the resistant allele itself. However, insecticide applications would select a similar haplotype containing the resistant allele. It has been demonstrated that the same resistant allele (either *ace-1*^*R*^ or *kdr*) spread among species of the *An. gambiae* complex in West Africa through introgression of the same allele^44^,^45^,^46^. Thus, we expect that the phenotypes associated with insecticide resistance observed in our strain are also true in natural mosquito populations.

In absence of *Plasmodium* infection, the main insecticide resistance mutations have affected differently the survival of *An. gambiae*. While no cost or advantage has been associated with the OP resistant allele *ace-1*^*R*^, in the tested conditions, the *kdr* resistant allele was associated with an increased survival (HR = 1.37, CI: 1.12–1.68), suggesting a selective advantage in insecticide free environment. In insect, variation in longevity could be the consequences of resource-based trade-offs and oxidative stress^26^. A decreased neuronal and behavioral excitability observed in mosquito and moth harboring the *kdr* mutation^24^,^47^ may be associated with a lower energy consumption that would benefit to survival. The higher survival rate observed in *kdr* mosquitoes could be also due to lower level of oxidative stress as unbalanced production of reactive oxygen species is detrimental according to the free-radical theory of ageing^48^,^49^.

In contrast, fecundity rate was reduced in both insecticide resistant strains compared to the susceptible strain Kisumu but no difference in the number of eggs per female was observed between mosquito strains. This lower fecundity rate indicated a fitness cost associated with both insecticide resistance alleles. In the field, *An. gambiae s.l.* commonly required two blood meals to mature and lay their first batch of eggs probably due to insufficient energetic reserves^40^,^41^. Depending on the teneral reserves accumulated during larval development, mosquito will use their first blood meal to refill their energetic stores instead of producing eggs^50^. The observed lower proportion of female developing eggs may be due to lower teneral reserves in insecticide resistant mosquitoes, as observed in *C. pipiens* bearing the *ace-1*^*R*^ resistant allele^51^. Teneral reserves are positively related to nutrition and negatively related to larval rearing temperature and density^52^,^53^. As rearing conditions were similar between mosquito strains in our experimental design, one hypothesis to explain the differences in teneral reserves is the impairment of food intake and digestion and energy storage associated with insecticide resistance in mosquito larvae. Another hypothesis is that variation in larval competition could be associated with insecticide resistance leading to difference in energy stored. Further evidence with direct measurement of energetic resources is needed to test these hypotheses.

After the second blood meal to complete the first gonotrophic cycle, a larger proportion of females produced eggs with no difference between Kisumu and Acerkis strains but Kdrkis still showed a lower fecundity rate. Overall, regardless of the number of blood meal taken to complete their first gonotrophic cycle, Kdrkis females had a significantly lower fecundity rate (69% ± 6.9, *p* < 0.001) compared to Kisumu (87.5% ± 8.4) but not Acerkis females (75% ± 7.8, *p* = 0.146). Therefore, we suggest that the *ace-1*^*R*^ allele may be associated with an energetic rather than a reproductive cost: lower teneral resources could be compensated with the first blood meal resulting in similar fecundity rate compared to the insecticide susceptible strain following subsequent blood meals. However, the *kdr* allele may be rather associated with a reproductive cost because additional blood meal did not abolish the reduction in fecundity rate on one hand, and on the other hand *kdr* mosquitoes showed an increased survival. According to Dao *et al*.^54^, reproduction can be influenced by courtship (*i.e.* male-female interaction), mating, quality of sperm and seminal fluid, egg development and oviposition. However, our data did not suggest a cost on egg development as the number of eggs produced was similar for all strains and all blood meals.

The effect of insecticide resistance on mosquito survival and fecundity has been also assessed in presence of infection. In presence of *Plasmodium* infection, *ace-1*^*R*^ resistant allele was associated with a cost on survival when compared to the susceptible allele, while survival of *kdr* individuals was similar to that of the susceptible ones. In contrast, *Plasmodium* infection did not influence the reproductive cost (*i.e.* the reduction of fecundity rate) associated with insecticide resistance.

*P. falciparum* infection did not affect mosquito survival in the reference susceptible strain. Under similar nutritional condition (2.5% glucose), *P. falciparum* infection cost in *An. coluzzii* was found to be dependent upon blood donor: no cost was detected on some feeding assays whereas cost on survival was detected on others^32^. Previous studies performed on field collected *An. gambiae* infected with *P. falciparum* did not reveal any survival cost^28^,^29^. However in the present study, *Plasmodium* infection was associated with a survival cost in both insecticide resistant strains when compared to the corresponding *Plasmodium* non-exposed strains. Resistant *An. gambiae* provides a new physiological environment to *P. falciparum* in which parasites could be less adapted compared to the ancestral insecticide susceptible host. Considering that the reduction in survival was only observed in infected mosquitoes, we suggested that the insecticide resistant alleles, or closely linked alleles, may interfere directly or indirectly with important factors of the immune system^55^ that would increase the cost of mounting an immune response against *P. falciparum*.

In addition, infection influenced the fecundity of *An. gambiae s.s.* independently of the mosquito strain considered. After one infectious blood meal, *P. falciparum* infection increased the fecundity rate. It is not clear how parasite infection would lead to this observation but it is assumed that immune-challenged mosquitoes would probably invest more in reproduction in order to increase their fitness if the cost of mounting an effective immune response is very high^56^. When females needed two blood meals to complete their first gonotrophic cycle, *Plasmodium* infection did not influence the proportion of females producing eggs but the number of eggs was lower compared to females non-exposed to *P. falciparum*. These results suggested that pre-existing *Plasmodium* infection had altered egg production or development and is consistent with field data, showing that *P. falciparum* infection is associated with a reduction of egg production in wild caught *An. gambiae*^30^. As mounting an effective immune response is expected to be costly, infected individual may resorb eggs to produce energy for maintenance of others physiological processes^57^ and could explain the reduction of eggs associated with *Plasmodium* infection.

Although our experimental design did not allow testing the relationship between variation in reproduction and survival in infected *An. gambiae* mosquitoes, the hypothesis that investment in reproduction reduces survival (trade-off hypothesis^58^) is consistent with the observed results on both resistant strains but not on the susceptible one. Indeed, *Plasmodium* infection reduced survival of both insecticide resistant strains but increased the proportion of female producing eggs. This is in contrast with the results obtained in the natural avian malaria system^33^ where infection of *C. pipiens* (resistant or susceptible to insecticides) with *P. relictum* resulted in a reduction in fecundity (number of eggs produced) and an increased survival. Although the trade-off hypothesis was not tested directly in our study, the discrepancy may rely on the experimental design (*i.e.* absence of oviposition site in our survival experiment) and in the nature of the vector-parasite interaction.

From an epidemiological point, it is unknown whether the combined effects of insecticide resistance and *Plasmodium* infection on survival and reproduction would impact on vector population dynamics and hence on the vectorial capacity. On one hand, increased survival associated with the *kdr* allele may lead to the spread of this allele in natural populations of mosquitoes even in absence of insecticide selective pressure. On the other hand, cost of infection on survival in insecticide resistant mosquitoes would strongly reduce the vectorial capacity for malaria^59^ because insecticide resistant vectors may not survive through the extrinsic incubation period.

Moreover, *Plasmodium* infected mosquitoes harboring the *ace-1*^*R*^ or the *kdr* allele have a reduced level of resistance to insecticides leading to a greater probability of dying after insecticide exposure than uninfected mosquitoes^37^. Rather than the simplistic assumption that insecticide resistance would undermine the control of vector-borne diseases, we provide evidence for more complex effects of insecticide resistance that make more difficult the prediction of its epidemiological impact. Understanding the influence of adaptive gene, not only at the species or population level, but at the ecosystem level (*i.e.* mosquito-parasite interaction) will provide crucial insight for the design of vector-borne disease control strategies.

The survival cost evidenced here suggests that insecticide resistance have the potential to reduce vectorial capacity while we previously showed that it increases vector competence^22^. This may have a great impact on transmission as considering the Ross-MacDonald equation of the vectorial capacity^60^, vector survival has a greater influence than vector competence. Prediction of transmission dynamics by mosquito vectors without including ecological interactions between parasites and vectors could lead to misleading results or wrong epidemiological outcomes. The impact of insecticide resistance and of malaria parasite infection appeared to be complex and therefore, its effect on others components of the vectorial capacity, like the mosquito biting behavior, needs to be assessed. In addition, there is increasing evidence for multiple insecticide resistance in *An. gambiae* that makes the prediction even more complex. Taking into account the ecological interactions between selected insecticide resistant alleles, mosquito and parasite life-history traits, and the environment (such as the presence of insecticides) would allow better evaluation of current vector control strategies and foster the development of new evolutionary-proof control measures.

## Material and Method

### Ethical Statement

All experiments were performed in accordance with the France and Burkina Faso guidelines and regulations and were approved by the Centre Muraz Institutional Ethics Committee under the ethical clearance number 003–2009/cE-cM. All human volunteers were enrolled after written informed consent from the participant and/or their legal guardians.

### Mosquito strains

Three reference strains of *Anopheles gambiae sensu stricto* (S molecular form) were used in this study. One is the reference insecticide susceptible strain Kisumu, collected in Kenya in 1953^61^. The two other strains were resistant to two distinct class of insecticide: the strain Acerkis, homozygous for the *ace-1* G119S mutation and resistant to organophosphates and carbamates (OP/CX), and the strain, homozygous for the *kdr* mutation and resistant to pyrethroids (PYR) and DDT. The insecticide resistant strains were obtained by introgression of the resistant mutations (*ace-1* G119S or *kdr-west* L1014F) allele into the Kisumu genome. The *ace-1* G119S allele was obtained from a sample of a resistant *An. gambiae* population collected in Bobo-Dioulasso, Burkina Faso in 2002^62^. The *kdr-west* allele has been obtained from pyrethroid resistant mosquitoes sampled in Kou Valley, Burkina Faso^13^. Kisumu and wild *An. gambiae* from Burkina Faso used for selection of the insecticide resistant alleles shared polymorphism for the chromosomal inversions 2Rb and 2La^63^,^64^. As the resistant alleles are located far from them^22^, these inversions are not expected to affect recombination at the selected loci due to low linkage disequilibrium^65^,^66^. However, it is known that selection of alleles conferring resistance may hitchhike neighboring polymorphisms and it is therefore expected that the strains differ in the regions flanking the introgressed alleles^44^. Insecticide resistant phenotype of all strains was regularly verified using the WHO standard vertical tube protocol^67^ with 0.1% bendiocarb and 4% DDT impregnated papers, and the presence/absence of the mutations were checked using the *ace-1* G119S and *kdr* diagnostic PCR^13^,^68^. Mosquitoes were kept under standard insectary conditions (27 ± 1 °C, 70 ± 8% RH and 12:12 light and dark photoperiod) in the same secure containment facility. Larvae were reared in the same condition at a fixed density (300 first instar larvae in 700 ml of water per tray) in local spring water and were fed *ad libitum* with Tetramin^®^Baby in order to reduce variation in larval growth rate and mosquito size at emergence. After emergence, adults were fed *ad libitum* on a 5% glucose solution and maintained in two to three 30 × 30 × 30 cm cages. For each experiment/replicate, pupae were collected and placed in a new cage during three consecutive days. One day prior to blood exposition, mosquitoes were starved by removing the glucose solution.

### P. falciparum experimental infection

Standard membrane feeding assays were performed as previously described^22^,^38^ with slight modification according to Sangare *et al*.^39^. Briefly, *P. falciparum* gametocyte carriers were selected by examining thick blood smears from children aged between five and eleven from two villages in southwestern Burkina Faso (Dandé and Soumousso, located 60 km north and 40 km south-east of Bobo-Dioulasso, Burkina Faso, respectively). Children with a gametocyte density of more than twenty per μl of blood were selected and a venous blood sample (8 ml) withdrawn. Blood serum was replaced with European naive AB serum to limit the potential effect of human transmission blocking immunity^69^. Reconstituted blood samples were divided in two batches. One batch was heated at 42 °C for 15 min to heat-inactivate gametocyte infectivity and was used as not infectious blood to produce the negative control for infection^39^. The other batch of blood was used without heat-treatment for infectious blood meal. Membrane feeders were filled with 500 μl of reconstituted blood and maintained at 37 °C by water jackets. Three to five day-old female mosquitoes of the three mosquito strains were allowed to feed concomitantly on infectious or heat-inactivated blood for up to 30 minutes through a Parafilm membrane. Two to three feeders were used for each mosquito strains in order to limit potential feeder effect. Unfed female mosquitoes were discarded and only fully fed mosquitoes were maintained. This procedure was repeated five times for the survival and the fecundity experiment, respectively, each feeding assay (*i.e.* replicate) using a different gametocyte-infected blood and different batches of each mosquito strain.

### Survival experiment

After blood feeding and isolation in individual plastic tubes (*Drosophila* vials 25 × 95 mm), females were allowed to feed daily on a cotton-imbibed with a 2.5% glucose solution. Mortality 24 h following blood feeding did not indicate any influence of mosquito genotypes nor *Plasmodium* gametocytes (data not shown) and were not included in the analysis in order to disregard feeding-associated mortality. From the second day after blood feeding, dead females were removed every eight hours (more or less one hour) and dissected until all females had died. Wings and midguts were removed from freshly dead female mosquitoes. No further blood meals were given during the course of the experiment and no oviposition substrates were provided.

### Fertility and fecundity experiments

After blood feeding on gametocyte-infected and control blood, distinct batches of females were isolated individually to lay eggs on a wet paper and were allowed to feed on a 2.5% glucose solution. Once they laid eggs, females were dissected to look for remaining eggs and wings and midguts were removed. Eggs on paper were counted under a binocular microscope. Females that did not lay eggs after the fifth day post-blood meal (PBM) were allowed to re-blood feed on a rabbit and were isolated to lay eggs. Females and egg papers were processed as above to measure wing length, and to count oocysts, eggs laid and non-laid eggs. Females that do not lay eggs after the 10^th^ day after the first blood meal (fifth day after the second one) were dissected to look for developed eggs in the ovaries and to measure wing length. Oocysts were not counted on these females to avoid underestimation as some oocysts may already have released sporozoites.

### Oocyst counting and measuring of wing length

Midguts were dissected in 0.4% mercurochrome stain and oocysts were counted under a light microscope on each individual female that took an infectious blood meal. The left wing of each female mosquito was cut and was pictured with a dissecting microscope (Leica EZ4D). Wing length was measured from the notch to the wing tip as previously described^70^. Two measures were performed independently using the ImageJ software (Wayne Rasband, rsb.info.nih.gov/ij/) and correlation between both indicated good agreements (R^2^ = 0.98), thus, the mean of the two measures was used.

### Blood meal size determination

In the survival experiment, blood meal size was quantified retrospectively by measuring the heamatin excreted from individual females in each *Drosophila* tube, as described by Briegel^71^. Heamatin excretion was dissolved in 1 ml of a 1% LiCO_3_ solution and the absorbance of the resulting mixture was read at 387 nm. Blank was obtained with LiCO_3_ solution alone to correct measures of each individual. Heamatin content was estimated by comparing with a standard curve made with porcine serum heamatin (Sigma-Aldrich).

### Statistical analyses

We examined the relationship between survival rate of mosquitoes and the presence of *P. falciparum infection* (categorical variable of two levels), insecticide resistance *strain* (categorical variable of three levels), *heamatin* content (proxy of blood meal size, numerical variable), *wing* length (proxy of body size, numerical variable) and the blood *donor* (categorical variable of five levels). Survival distributions of the three *An. gambiae* strains either infected or control (unexposed) were computed using the Kaplan-Meier method stratified with respect to the *donor* variable^72^ using the LIFETEST procedure. The differences between survival distributions were estimated using the asymptotic Log-Rank Test^73^ taking into account the influence the blood donor. The stratification by the variable *donor*, corresponding to each experimental infection, allowed having a different baseline hazard function, while the coefficients of the remaining covariates are assumed to be constant across strata.

Cox proportional hazard regression (PHREG procedure) was used to identify whether any of the five following explanatory variables: insecticide resistance *strain*, *oocyst burden*, blood *donor*, *heamatin* content (blood meal size) and *wing length*, could explain the observed variation in the survival of infected mosquitoes. Maximal models included all main effects and all interactions between *genotypes*, *heamatin*, *wing length* and the *oocyst count* variables, and implemented by stratifying with respect to *donor*. First, the best distribution of the response variable (Weibull distribution) was chosen based on the lowest Akaike Information Criterion (AIC). AIC-based stepwise selection has been performed and significance of variable of the minimum model was assessed by analyzing deviance.

Analysis of the fecundity was based on the two different response variables measured over 5 feeding assays with distinct blood donors: *fecundity rate* (proportion of female producing eggs) and *fecundity* (number of eggs) for females that produced at least one egg. Data was analyzed using a generalized mixed-effect model with a Poisson distribution for the fecundity or a binomial distribution for the fecundity rate using the GLIMMIX procedure. The maximal models included the following explanatory variables and their interactions: insecticide resistance *strain*, *P. falciparum* infection, *wing* length and the blood *donor*. The random structure with the *donor* gives the lowest AIC. This procedure performs a type III hypothesis for the fixed effect variables and computes the F-statistic based on Satterthwaite’s approximation. Mean percentage of egg-laying females (fecundity rate) and mean number of eggs per female (fecundity) were computed. Post-hoc tests (Lsmeans statement within the GLIMMIX procedure) were carried out to assess differences between estimates, and Bonferroni corrections were applied for multiple comparisons. All statistical analyses were performed using statistical analysis software (SAS Institute Inc., Cary, NC).

## Additional Information

**How to cite this article**: Alout, H. *et al*. Interactive cost of *Plasmodium* infection and insecticide resistance in the malaria vector *Anopheles gambiae.* *Sci. Rep.* **6**, 29755; doi: 10.1038/srep29755 (2016).

## Supplementary Material

Supplementary Information

## Figures and Tables

**Table 1:** Hazard ratio associated with insecticide resistance alleles and *P. falciparum* infection in *An. gambiae s.s*. survivorship.

Insecticide resistance	Insecticide resistance	Median survival	Hazard ratio	Lower 95%CI	Upper 95%CI	Log-rank χ^2^	*p*-value
	Unexposed Kisumu*	13	—	—	—	14.48	p < 0.001
	Unexposed Acerkis	14	0.957	0.779	1.175		
	Unexposed Kdrkis	15	**1.373**	1.120	1.682		
	Infected Kisumu*	13	—	—	—	32.86	p < 0.001
	Infected Acerkis	12	**0.552**	0.441	0.692		
	Infected Kdrkis	13	0.898	0.722	1.116		
***P. falciparum*** **infection**	***P. falciparum*** **infection**	**Median survival**	**Hazard ratio**	**Lower 95%CI**	**Upper 95%CI**	**Log-rank χ**^**2**^	***p*****-value**
	Unexposed Kisumu*	13	—	—	—	1.540	0.210
	Infected Kisumu	13	0.897	0.716	1.122		
	Unexposed Acerkis*	14				49.45	p < 0.001
	Infected Acerkis	12	**0.518**	0.420	0.638		
	Unexposed Kdrkis*	15				39.26	p < 0.001
	Infected Kdrkis	13	**0.587**	0.481	0.715		

Hazard ratio was estimated by comparing survivorship with that of a reference (indicated by an asterisk) and significance was tested using post-hoc tests with a Bonferroni correction.

**Table 2:** Fecundity rate and number of eggs produced of infected and control mosquitoes for each *Anopheles gambiae* strain.

Insecticide resistance		One Blood Meal				Two blood Meals			
Infection group	Strain	Fecundity rate	*p*-value (compared to Kisumu)	Fecundity	*p*-value (compared to Kisumu)	Fecundity rate	*p*-value (compared to Kisumu)	Fecundity	*p*-value (compared to Kisumu)
Control	Kisumu	0.490 (0.072)	—	49.50 (6.79)	—	0.668 (0.080)	—	63.85 (8.61)	—
	Acerkis	0.278 (0.059)	**0.001**	61.14 (9.39)	0.159	0.644 (0.074)	0.762	61.92 (8.00)	0.828
	Kdrkis	0.277 (0.060)	**0.001**	50.73 (8.06)	0.874	0.455 (0.082)	**0.013**	58.92 (8.23)	0.579
Infected	Kisumu	0.594 (0.075)	—	51.49 (7.47)	—	0.657 (0.081)	—	50.68 (7.09)	—
	Acerkis	0.405 (0.075)	**0.045**	47.34 (7.11)	0.585	0.706 (0.070)	0.518	45.13 (5.71)	0.388
	Kdrkis	0.339 (0.073)	**0.002**	64.76 (10.74)	0.164	0.442 (0.076)	**0.008**	47.94 (6.35)	0.707
***P. falciparum*** **infection**		**One Blood Meal**				**Two blood Meals**			
**Strain**	**Infection group**	**Fecundity rate**	***p*****-value (compared to Control)**	**Fecundity**	***p*****-value (compared to Control)**	**Fecundity rate**	***p*****-value (compared to Control)**	**Fecundity**	***p*****-value (compared to Control)**
Kisumu	Control	0.490 (0.072)	—	49.50 (6.79)	—	0.668 (0.080)	—	63.85 (8.61)	—
	Infected	0.594 (0.075)	0.152	51.49 (7.47)	0.784	0.657 (0.081)	0.903	50.68 (7.09)	0.119
Acerkis	Control	0.278 (0.059)	—	61.14 (9.39)	—	0.644 (0.074)	—	61.92 (8.00)	—
	Infected	0.405 (0.075)	0.055	47.34 (7.11)	0.086	0.706 (0.070)	0.356	45.13 (5.71)	**0.007**
Kdrkis	Control	0.277 (0.060)	—	50.73 (8.06)	—	0.455 (0.082)	—	58.92 (8.23)	—
	Infected	0.339 (0.073)	0.345	64.76 (10.74)	0.152	0.442 (0.076)	0.861	47.94 (6.35)	0.141

Estimates were compared between strains to assess the effect of insecticide resistance and between control and infected for each strain separately. Difference was tested using post-hoc tests with a Bonferroni correction.
