# Supplementary material for: Interactive cost of Plasmodium infection and insecticide resistance in the malaria vector Anopheles gambiae
Source: Sci Rep. 2016 Jul 19;6:29755. doi: 10.1038/srep29755 (PMC4949420; doi:10.1038/srep29755)
Supplement: Supplementary Information [file srep29755-s1.pdf]

Interactive cost of *Plasmodium* infection and insecticide resistance in the malaria vector *Anopheles gambiae*

Haoues Alout*, Roch K. Dabiré, Luc S. Djogbénou, Luc Abate, Vincent Corbel, Fabrice Chandre, Anna Cohuet

Supplementary information

Results

Genotyping of SNPs in perfect linkage disequilibrium with the *para* locus

SNP genotyping of individual mosquito indicated that all Kdrkis individuals shared the same SNPs while polymorphism was detected among individual of the Kisumu strain. This indicated that the same *kdr*-bearing haplotype containing at least the *para* (with the *kdr* mutation) and ClipC9 loci has been selected by insecticide exposure during the process of introgression.

ClipC9 exon 1		Kisumu	Kdrkis
207 [A/G]	252 [T/G]		
A/A	T/T	6	15
A/A	G/G	0	0
G/G	T/T	0	0
G/G	G/G	0	0
A/A	T/G	0	0
A/G	T/T	0	0
A/G	T/G	9	0
**Total**		**15**	**15**

para-ClipC9 SNP	Kisumu	Kdrkis
51 [C/T]		
C/C	3	0
T/T	2	16
C/T	11	0
**Total**	**16**	**16**

Supplementary table. Genotype at selected SNP in the insecticide susceptible strain Kisumu and the pyrethroid resistant strain Kdrkis. SNPs were selected based on their location relative to the *para* locus (potentially bearing the *kdr* mutation) and the ClipC9 locus (potentially involved in immunity and linked to *para*, Mitri et al. 2015). Two SNPs are located in the exon 1 of ClipC9 and one is located between the *para* and ClipC9 loci.

Material and Method

Individual mosquitoes from the Kisumu and the Kdrkis strains were genotyped using two SNPs in perfect linkage disequilibrium with the *para* locus: one located between the *para* and the ClipC9 loci (AGAP004711, Mitri et al. 2015), the other located in the exon 1 of ClipC9 (AGAP004719). Sixteen individual from each strain were subjected to genomic DNA extraction using the Qiagen DNeasy Blood & Tissue kit (Valencia, CA) according to the manufacturer instructions. Amplification was carried out with the following primers sets, para-clipF: TATCGGTCGGTCGAGTCATT; para-clipR: ATTACATGCTGCACATCCGG; ClipC9ex1F: GGTGCAGTAAGAAGGCCCAT; ClipC9ex1R: CGCATTGCATCGATTCAGCA. PCR reactions were performed using the following cycling conditions: an initial denaturation at 95°C for 2 min, 35 cycles at 95°C for 30 s, 55°C for 30 s and 72°C for 1 min, and a final extension at 72°C for 5 min. Amplicons were purified using the Agencourt AMPure PCR DNA purification kit (Agencourt, Beverly, MA) and subjected to direct bidirectional sequencing with the original primers. Sequencing reactions were performed using the BigDye Terminator v3.1 mix and the sequencing products were cleaned with CleanSEQ magnetic beads (Agencourt, Beverly, MA) and analyzed on a 3130xl genetic analyzer (Applied Biosystems, Foster City, CA, USA).

Sequence of SNP between *para* and clipC9:

TATCGGTCGGTCGAGTCATTTAGAAATCAAGAGAAGGACGTGCTAGTTGC**[T/C]**A

CTGACGTCGCATCTAAAGGTTTGGATTTTCCGGATGTGCAGCATGTAAT

Sequence of amplified ClipC9 exon 1:

GGTGCAGTAAGAAGGCCCATTAATATACGGGTATTGGAAGGAAATAGTTGTGAC

ACTCCTCAAGTTATCGGTGGAAAGTGCATGAATATATCTTTATGTGATCCAGCTTT

TGTGCACTCCATAGCCTATCAAGAGCATACCCCGGTGTGTCAACAAAATGCCTTC

TACAGAGTGATTTGCTGTCAACCGTTTCTTGATTTTTGCGA**[A/G]**AATAGTAAACA

ATTTCAAATCATGCACGGCATTGAAGCAGAACC**[T/G]**GGAATGTTTCCCCACCTTG

CTAGATTAGGGTTGAAAAGTGAAGAGGATGGGATTGCTTGGACATGCAGTGCGA

ATATTATTTCCGAACGATTTCTCCTTACCGCTGCGCATTGCAATCCGGTGAACATT

GCGGGACTCGGTTGTGCTGAATCGATGCAATGCG
